# Realizing human rights in rural Punjab of India: a study of enforcement of selected human rights

**DOI:** 10.3389/fsoc.2025.1619603

**Published:** 2025-09-16

**Authors:** Puneet Pathak, Vagisha Nandini, Deepesh Yadav, Sukhwinder Kaur, Rajinder Kumar Sen, Shanti Riang

**Affiliations:** ^1^Department of Law, Central University of Punjab, Bathinda, Punjab, India; ^2^Department of Hindi, Central University of Punjab, Bathinda, Punjab, India

**Keywords:** human rights, local self-governance, Gram Panchayats, right to education, right to health, right to political participation

## Abstract

The realization of human rights assumes great significance in the context of India, the largest democracy in the world. Being a state party to binding international human rights covenants, India is obligated to take measures to ensure the realization of human rights by its citizens. With over 64% of its population residing in the villages, the panchayati raj institutions assume a crucial role in the realizing of human rights, considering their proximity to the rural population. The study's objective is to analyze the status of realization of selected human rights in terms of the ‘respect, protect, fulfill and promote' framework, i.e., health, education and political participation in the selected Gram panchayats of Indian Punjab. The study adopted a quantitative approach, and primary data were collected through survey questionnaires from members of selected Gram Panchayats and beneficiaries availing the benefits of related governmental schemes. The study offers insights into the degree to which the state obligations regarding the selected human rights are being upheld and how the variation can be explained. The result revealed inconsistencies between the claims of panchayat members and the beneficiaries' experiences. The study exposed deficiencies in infrastructure and service delivery across the rights. The study recommends improving awareness and satisfaction regarding the National Rural Health Mission (NRHM) and the Samagra Shiksha Abhiyan schemes for fulfilling the rights to health and education. Enhanced efforts are needed to promote these rights through regular awareness campaigns and discussions at Gram Sabha meetings. Furthermore, there is a need to strengthen campaigns for electoral participation, ensuring consistent and well-communicated Gram Sabha meetings, and active facilitation of the political involvement of marginalized groups for the effective realization of the right to political participation.

## Introduction

Human rights are those that people naturally hold by virtue of their existence (Tasioulas, 2013). Every person is entitled to human rights, regardless of their “race, color, sex, language, religion, political or other opinion, national or social origin, property, birth, or other status,” according to Article 2 of the Universal Declaration of Human Rights (UDHR) (Adeola, 2023). There are three generations into which human rights fall (Ife et al., 2022). The right to life and liberty, the right to property, the right to vote, and other civil and political rights are known as first-generation human rights, or “blue rights,” and they date back to Magna Carta in 1215 (Cianitto, 2024). Economic, social, and cultural rights like the right to food, shelter, health care, and social security are second-generation rights, sometimes called “red rights” (van Rensburg and Naudé, 2007). The third generation of rights, sometimes called "green rights”, are solidarity rights that include rights like the right to self-determination, development, and a healthy environment (Glazewski, 1991). Human rights can be protected at the national level by being enshrined in the Constitution and pertinent laws, while they are acknowledged internationally by treaties and conventions (Buergenthal, 2006). By adopting General Assembly resolution 217 A (III) on December 10, 1948, the United Nations General Assembly declared the UDHR as a “*common standard of achievement for all peoples and all nations”* (Hannum, 1998). UDHR, a fundamental document supplemented by two binding international human rights covenants, recognizes civil and political rights and economic, social, and cultural rights (Ssenyonjo, 2010).

### Respect, protect, fulfill and promote framework

The “respect, protect, and fulfill” framework for the realization of human rights was devised to move away from the dichotomy of negative and positive rights (Karp, 2020). Civil and political rights were considered as negative rights; to guarantee such rights, the state only needed to refrain from actions infringing such rights and was not obliged to provide any services for the same (Joseph, 2010). Social, economic and cultural rights such as the right to food, the right to shelter, the right to safe drinking water, etc., were considered positive rights as it was felt that to guarantee these rights, state action was needed (Chapman, 1996). However, it was strongly felt that it was necessary to move away from this distinction (Hirschl, 2000). In his seminal book ‘Basic Rights', Henry Shue argued that every basic right had three types of correlative duties: duties to avoid depriving, duties to protect from deprivation and duties to aid the deprived (Shue, 2020). These three duties laid down the foundation of the respect, protect and fulfill framework in the works of Philip Alston and Asbjørn Eide (Bodig, 2012). The framework was formally adopted for the first time in the Maastricht Guidelines on Violations of Economic, Social and Cultural Rights where it was stated that “*…like civil and political rights, economic, social and cultural rights impose three different types of obligations on States: the obligations to respect, protect and fulfill”* (Dankwa et al., 1998). The Maastricht guidelines laid down that “*…The obligation to respect requires States to refrain from interfering with the enjoyment of economic, social and cultural rights. …The obligation to protect requires States to prevent violations of such rights by third parties. …The obligation to fulfill requires States to take appropriate legislative, administrative, budgetary, judicial and other measures toward the full realization of such rights*” (Flinterman, 1997). It further stated that the state parties' failure to perform either of these obligations constituted a violation of the right concerned. The “respect, protect and fulfill” framework was prominently used with the right to food by the UN Committee on Economic, Social and Cultural Rights in its General Comment 12 on the right to food (Karp, 2020). Afterwards, it was also incorporated by the Committee in General Comments 13 and 14 on the right to education and health (Nampewo et al., 2022). The influence of the ‘respect, protect and fulfill' framework can also be seen in philosophy, political science, public international law, research and advocacy of human rights organizations (Karp, 2020). There have been attempts to refine this tripartite framework in which van Hoof proposed a fourth category, i.e. obligation to promote (Koch, 2005). Eide also proposed a duty to facilitate after the duty to fulfill (Eide, 2010). Steiner and Alston reworked the framework to propose five levels of obligations: respect, protect, facilitate, provide, and promote (Sengupta et al., 2005). However, the tripartite ‘respect, protect and fulfill' framework has gained the most traction in the contemporary human rights discourse (Mégret, 2020). It is an established doctrinal tool for interpreting the International Covenant on Economic, Social and Cultural Rights and a conceptual feature of all human rights (Koch, 2009).

The framework captures the entire spectrum of responsibility for all human rights and demolishes the binary divide of so-called positive and negative rights (Koch, 2005). The framework thus proposes that all rights, whether civil and political in nature or economic, social or cultural, in order to guarantee any of such rights, the state has three sets of obligations necessarily attached to them (Koch, 2003). The obligation to respect thus enjoins the state from interfering in the exercise of the right. In contrast, the obligation to protect enjoins the state to prevent violations from third parties. In contrast, the obligation to fulfill requires the states to ensure the provision of resources and outcomes of policies.

### Panchayati Raj institutions

Political, economic, and administrative decentralization are essential for localizing democracy and safeguarding human rights, according to the United Nations Human Rights Council (Friis-Hansen and Kyed, 2009). Gandhi also advocated decentralized democracy as it could ensure people's active participation in governance (Rajasekhar, 2021). Local self-government refers to the local body or authority's administration and oversight of local affairs. Local self-governance (LSG) institutions serve as the foundation of democracy in India (Jha, 2018). Participation in the governance and administration of these institutions offers individuals substantial exposure to both political and social dimensions (Huntington and Nelson, 2013). Rural local self-governance (RLSG) refers to a system of governance in rural areas where representatives are elected by village residents (Singh and Murari, 2019). RLSG functions through the institutions of Gram Panchayats, Block Samitis and Zila Parishad (Dutta and Ghosh, 2006). These entities operate as proactive agents of the state in preserving and furthering the essential human rights of rural communities (Mathew, 2003). Local governments must actively participate in respecting, protecting, fulfilling and promoting fundamental human rights if the state is to fulfill its obligations under the international human rights law (Durmuş, 2020). As a result, there is a clear need for research on implementing core human rights through RLSG among rural populations.

In 1992, the 73rd and 74th Constitutional Amendments established LSG's constitutional standing in India (Mathew, 1994). According to World Bank estimates, approximately 64% of India's population lives in rural areas (Sekher, 2012). The Panchayati Raj Institution (PRI) oversees rural local self-government in India (Datta, 2013). India's first Prime Minister, Jawahar Lal Nehru, coined the word ‘Panchayati Raj' while inaugurating the Nagaur Panchayat in Rajasthan on 2 October 1959 (Mathew, 2021). PRIs function at the village, intermediate (block), and district levels (Babu, 2021). There are an estimated 2,55,487 Village Panchayats at the village level, 659 District Panchayats at the district level and 6,829 Intermediate Panchayats at the block level (Meenu, 2022). According to the Reserve Bank of India's 2024 Report on Finances of Panchayati Raj Institutions, 31,87,320 elected PRI representatives work at all three levels in India (Sasidhar et al., 2024). Panchayats' power, authority, and duties are outlined in Article 243G of the Indian Constitution (Dube and Padalia, 2002). In addition to creating plans for social justice and economic growth, the Panchayats are also in charge of implementing the schemes entrusted to them (Singh, 2001).

The current study uses the “respect,” “protect,” “promote,” and “fulfill” framework to examine how the right to health, the right to education and the right to political participation are being realized in selected Gram Panchayats of Indian Punjab. Panchayats can significantly reduce human rights violations by encouraging human rights education and eliminating illiteracy at the village level (Mathew, 2003).

The study had two objectives. The first research objective was to understand the theoretical framework for realizing human rights. The second research objective was to analyze the status of realization of selected human rights, i.e., right to health, right to education and right to political participation in terms of the ‘respect, protect, fulfill and promote' framework in the selected panchayats of Indian Punjab.

## Methods

The present study adopted a quantitative approach to study the realization of three human rights, viz., right to health, right to education and right to political participation in selected gram panchayats of Punjab. Primary data was collected from members of Gram Panchayats and beneficiaries. The status of rural local self-government toward realizing human rights was studied quantitatively.

### Area of the study

The study was confined to the seven Gram Panchayats (Ghudda, Jai Singh Wala, Jhumba, Mann, Badal, Baho Yatri, Guru Ke) in Bathinda district of Punjab state. The Central University of Punjab, Bathinda, Punjab, adopted these Gram Panchayats.

#### Sample

The sample consisted of 70 panchayat members and 140 beneficiaries availing the government schemes, as shown in Table 1. The seven Gram Panchayats were coded from P1 to P7 as shown in Table 2.

**Table 1:** Sample.

**Panchayat members**	**Beneficiaries**	**Total sample**
(10 elected representatives from each panchayat)	(20 from each panchayat availing Govt. schemes relating to selected human rights)	
70	140	210

**Table 2:** Coding.

**Coding**	**P1**	**P2**	**P3**	**P4**	**P5**	**P6**	**P7**
Panchayat	Ghudda	Badal	Jhumba	Baho Yatri	Jai Singh Wala	Guru Ke	Mann

#### Tools

The tools employed for data collection/analysis:

1. Survey Questionnaire for Panchayat Members
2. Survey Questionnaire for Beneficiaries

The survey questionnaire for the panchayat members and beneficiaries contained 11 questions for each right, i.e., right to health, right to education and right to political participation, thus every respondent was asked a total of 33 questions. These eleven questions were designed according to the “respect,” “protect,” “promote,” and “fulfill” framework used worldwide to measure the realization of human rights. Accordingly, for the right to health and education, there were two questions each for the obligation to “respect”, three for the obligation to “protect”, four for the obligation to “fulfill” and two for the obligation to “promote”. Regarding the right to political participation, out of 11 questions, four were intended to measure “respect”, two for “protect”, three for “fulfill” and two for “promote”. A simple statistical analysis using averages and percentages was used for analyzing quantitative data.

Indicators for measuring the realization of the right to health

H1 awareness about the National Rural Health Mission
H2 awareness about the role of the elected panchayat representative in the National Rural Health Mission
H3 availability of medicine and quality services at the primary health center
H4 inspecting health officials‘ attendance and supervising health care providers' work, like ASHA and ANM.
H5 reviewing the maternal death/neonatal death/child death in the Gram Panchayat and identifying actions for the future.
H6 taking measures for the infrastructure development of primary health centers/sub-centers in the Panchayat.
H7 maintaining and monitoring overall cleanliness in villages to combat malaria, water-borne diseases, and vector-borne diseases.
H8 ensuring the effective functioning of the Village Health Sanitation and Nutrition Committee (VHSNC)
H9 ensuring regular immunization of pregnant and lactating women and children
H10 running awareness campaigns about the National Rural Health Mission
H11 raising issues related to health in the Gram Sabha meeting

Indicators for measuring the realization of the right to education

E1 awareness about the Samagra Siksha Abhiyan (SSA) Scheme
E2 awareness about the role of the elected panchayat representative under SSA
E3 measures to ensure equal opportunities to access school education for every section of students
E4 measures to ensure non-discrimination among children from backwards sections, minorities, females and disabled groups in school
E5 measures to ensure the safety of female students and school staff on the school campus
E6 ensuring the existence of government primary schools within 1 km and government upper primary schools within 3 km of nearby houses of children in a panchayat
E7 ensuring the distribution of free textbooks and school uniforms to all beneficiaries
E8 ensuring that the schools are equipped with modern infrastructural facilities (classroom, playground, benches and computer education), safe and clean drinking water facilities, and sanitation facilities
E9 ensuring separate toilets for female, male and disabled students
E10 running awareness campaigns about Samagra Shiksha Abhiyan
E11 organizing any special gram sabha meeting for education

Indicators for measuring the right to political participation

PP1 making campaigns for voting, joining a political party, or standing for elections
PP2 regularly attending Gram Sabha meetings
PP3 facilitating the process of contacting public officials
PP4 allowing and facilitating peaceful protest in the Panchayat
PP5 making any special campaign for SC/ST/Women/Minorities/Lower caste/ other marginalized people to participate in voting, joining a political party, or standing for elections
PP6 making any special campaign for SC/ST/Women/Minorities/Lower caste/ other marginalized people to participate in the Gram Sabha Meeting
PP7 regular conduct of Gram Sabha Meetings
PP8 prior notice about Gram Sabha Meeting
PP9 implementation of resolutions adopted in the meetings of the Gram Sabha
PP10 running of campaigns for people to actively and regularly participate in Gram Sabha meetings for public policy making
PP11 raising of issues related to public participation in the Gram Sabha meeting

The data collection tools employed in the present study were created using the tripartite classification of state obligations, i.e., the “respect, protect, fulfill” framework and the obligation to “promote” proposed by van Hoof. Thus, the survey questionnaires for each right were prepared per the indicators linked to the obligations of respect, protect, fulfill and promote. Even though the meanings ascribed to these four obligations are not set in stone and are subject to constant reinterpretation, the data collection tools in the current study employ this framework as per the traditional meaning in the Maastricht guidelines on violations of economic, social and cultural rights.

### Limitation

The study has some potential limitations. Since it was conducted in only seven Gram panchayats in the Bathinda district of Punjab, the findings may not be generalizable to other regions or states in India. Additionally, as the data was self-reported, there is a possibility of bias in the responses.

## Findings

### Status of realization of the right to health

**Figure 1:**
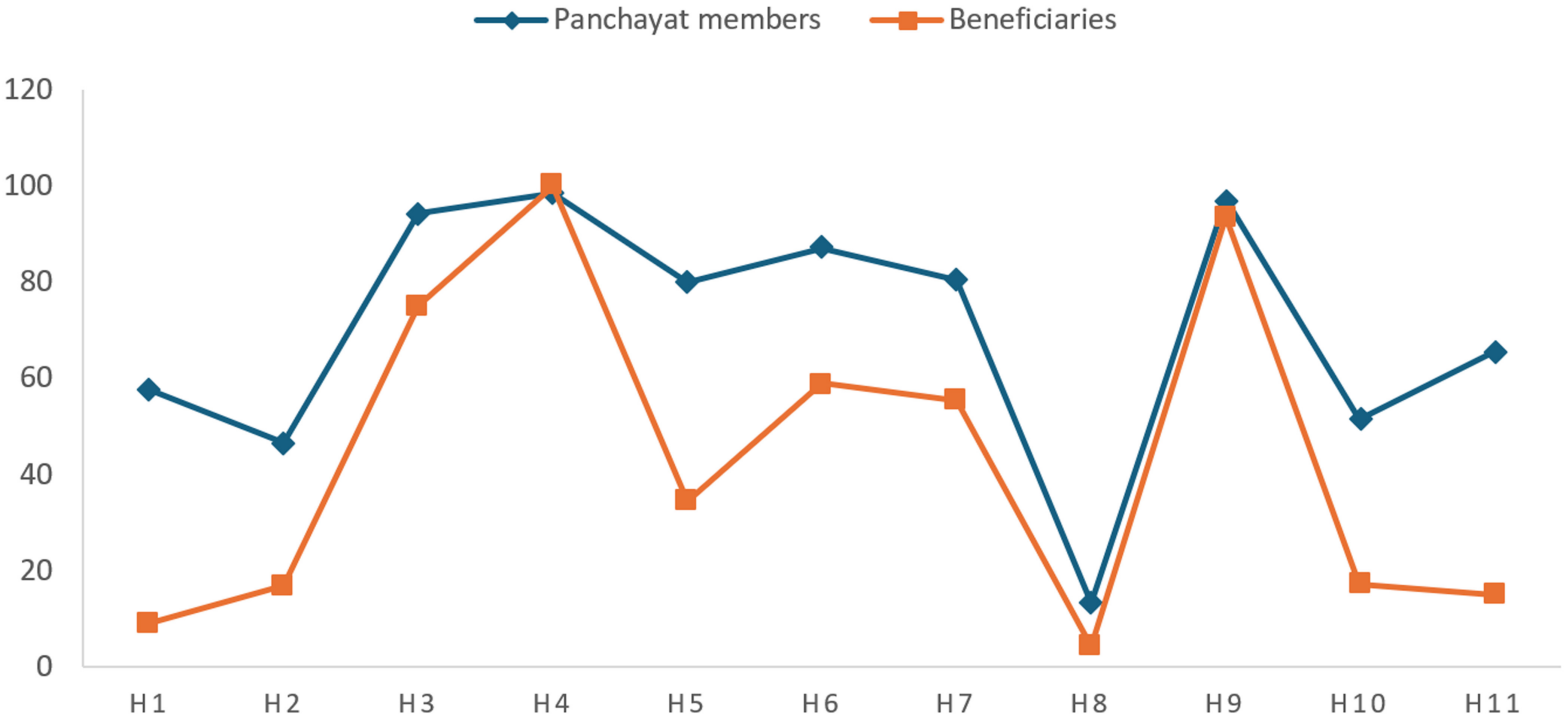
Status of the right to health.

The survey questionnaire administered to the panchayat members and the beneficiaries contained 11 questions related to the right to health (H). H1 and H2 were designed to measure the “respect” aspect, whereas H3, H4 and H5 were intended to see whether the right to health was being protected. H6, H7, H8 and H9 were designed to measure the aspect of fulfillment of the right. H10 and H11 dealt with the obligation to “promote”. Figure 1 compares the average responses of panchayat members and beneficiaries to the question related to the realization of the right to health through the National Rural Health Mission scheme.

Figure 1 illustrates a lack of awareness about the National Rural Health Mission scheme [H1] and the role of elected representatives regarding the same [H2] in both the panchayat members and the beneficiaries. The analysis of responses of H1 and H2 indicates a need for the state to improve the obligation to “respect” the right to health by educating the panchayat members about NRHM and their duties under the scheme.

Research has shown that the healthcare schemes do not reach people with low incomes owing to PRIs' inefficient and non-participatory role in decision-making (Nanjunda, 2020). There is a need to raise healthcare awareness and simplify the complicated procedures in such schemes. Studies have reported that most of the population is unaware of the different services under NRHM, which leads to inadequate utilization of the services available under the scheme (Ray, 2014). Lacunae regarding awareness and utilization of components of health services under NRHM continue to persist even after the launch of the third phase of NRHM (Choudhary et al., 2019).

The panchayat members actively supervise the health centers to ensure the availability and quality of services, as evidenced by their 94.28% response [H3]. The beneficiaries support this, as 75% agree that medicines and quality services are available at the primary health care center [H3]. The panchayat members (98.57%) and the beneficiaries (93.57%) are also unanimous in their opinion about the regularity of health officials and health care workers such as ASHA and ANM [H4]. While 80% of the panchayat members believe that the Gram Panchayat works to address local health issues [H5], the beneficiaries refute this, as only 34% agree. Gram Panchayats can potentially transform the state of health of the rural populace, and the studies confirm this (Mitra et al., 2014). The responses to H3, H4 and H5 show that the right to health is substantially protected at the panchayat level.

The panchayat members and beneficiaries also differ on the infrastructure development of primary health centers in the panchayat [H6] and the efforts to ensure cleanliness in villages to combat diseases [H7]. While 87.14% of panchayat members affirm the fulfillment of health rights by providing appropriate infrastructure for medical facilities, only 59.05% of the beneficiaries concur with this assessment. The responses of the panchayat members (13.33%) and the beneficiaries (4.28%) indicate alarming unawareness regarding the functioning of the Village Health Sanitation and Nutrition Committee (VHSNC) [H8]. The panchayat members and beneficiaries report almost 100 per cent immunization [H9], indicating that routine immunization of pregnant women and children is undertaken in the panchayats. The right to health is being fulfilled at the panchayat level, as the responses of the panchayat members are corroborated mainly by those of beneficiaries. However, the discrepancy between the reactions of the panchayat members and beneficiaries indicates that the infrastructure and medical facilities available to beneficiaries are inadequate. While panchayat members reported satisfactory health services, the poor response of beneficiaries points to their dissatisfaction with the availability and quality of medical care. Also, the panchayat members and beneficiaries' unawareness of the Village Health Sanitation and Nutrition Committee is a grave issue. The lack of awareness about VHSNC has been previously documented. Research has shown that panchayat members are still in the dark about these committees and have yet to receive any training regarding the operation of VHSNC (Malviya et al., 2014). Research has also shown that a lack of attention hinders the effectiveness of VHSNC, improper conduct of PRI officials, irregular sessions and a lack of funding (Kumar and Mishra, 2016).

The panchayat members and beneficiaries also disagree on implementing awareness programs about the National Rural Health Mission[H10] and raising the health issues in the Gram Sabha meeting [H11]. Since the obligation to “respect” is interlinked with the obligation to “promote”, we see that the impact of low scores in H1 and H2 is also reflected in H10 and H11, as shown in Figure 1. Thus, responses to H10 and H11 indicate that the state is lagging in its obligation to “promote”, and there is an urgent need to raise awareness about the health services available under NRHM. Research demonstrates that effective health promotion in rural areas involves comprehensive strategies, including infrastructure development, community engagement, and health education (Rifkin, 2009). Promotion must be multifaceted to be effective.

For ensuring the realization of the right to health, the panchayat members must be aware of their roles and duties as their engagement has been seen to be linked with improved attendance of health officials, availability of medicines, infrastructure and quality of services (Srivastava et al., 2016). The engagement of panchayat members in village health, sanitation, and nutrition committees has led to increased availability and consistency of healthcare practitioners at health centers (Kumar et al., 2016). Research also confirms that involvement of panchayats can significantly improve health indicators and raise public awareness about health initiatives (Trivedi et al., 2022).

### Status of realization of the right to education

**Figure 2:**
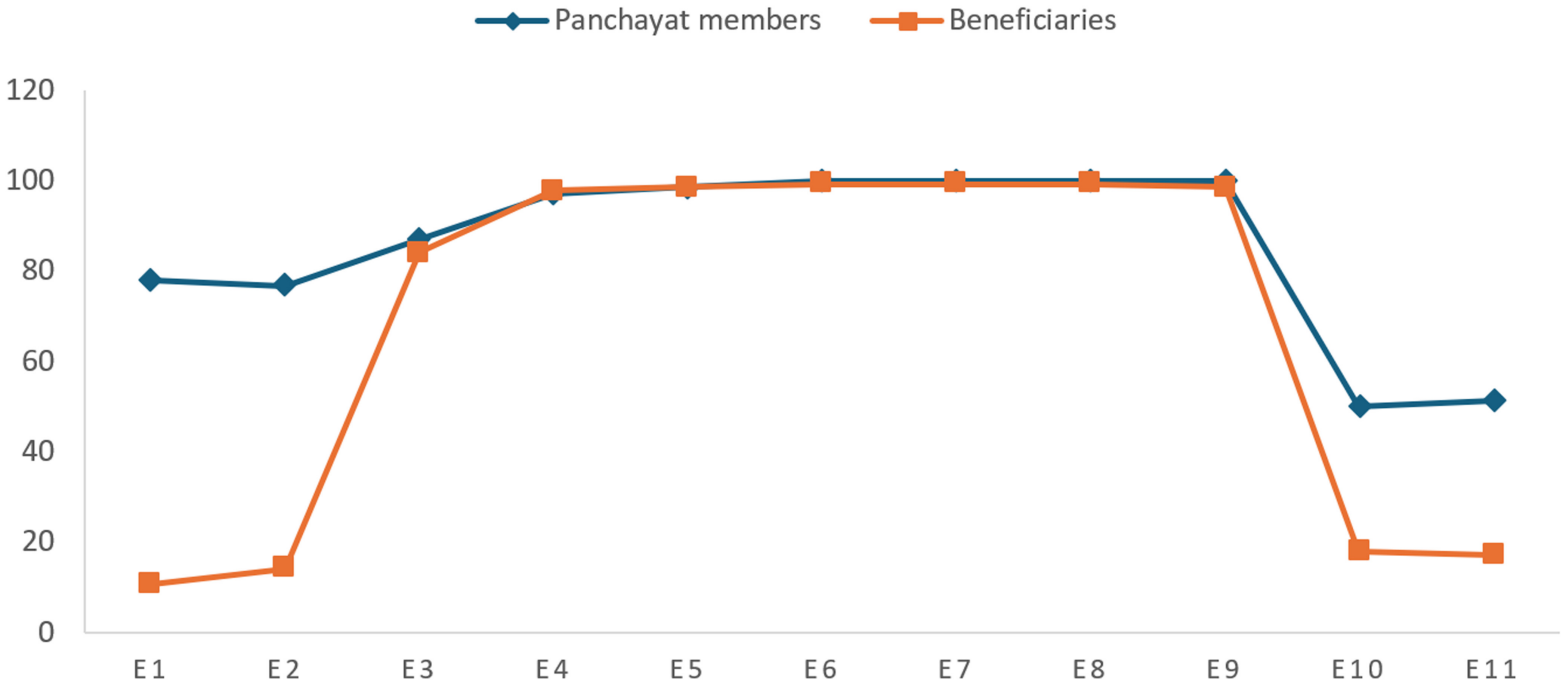
Status of the right to education.

The survey questionnaire administered to the panchayat members and the beneficiaries contained 11 questions related to the right to education (E). E1 and E2 were designed to measure the “respect” aspect, whereas E3, E4 and E5 were intended to see whether the right to education was being protected. E6, E7, E8 and E9 were designed to measure the aspect of fulfillment of the right. E10 and E11 dealt with the obligation to “promote”. Figure 2 compares the average responses of panchayat members and beneficiaries.

Figure 2 shows a stark difference between the responses of panchayat members and beneficiaries regarding awareness of the Samagra Shiksha Scheme [E1] and the role of panchayat members under the said scheme [E2]. While almost 80 per cent of panchayat members demonstrate awareness about the scheme, the beneficiaries' responses are dismal. Changing the scheme's name from Sarva Shiksha to Samagra Shiksha may be the cause behind the poor response of beneficiaries. Thus, the beneficiaries need to be educated about the scheme and the role of panchayat members. Literature supports these observations, highlighting that rural education often faces challenges related to infrastructure and resources but benefits from a community's recognition of its importance (Drèze and Sen, 2013). Studies show that community involvement in education can enhance respect for educational rights and improve school conditions (Sinha, 2005).

The responses of the panchayat members are substantially corroborated by those of the beneficiaries for all the questions designed to measure obligation to ‘protect' the right to education. Both classes of respondents unanimously agree that there are equal opportunities to access school education for every section of students [E3]. The outstanding responses of the panchayat members are also substantiated by the beneficiaries when it comes to the issue of non-discrimination among children from backwards sections, minorities, females and disabled groups in school [E4] and safety of female students and school staff in the school campus [E5]. Hence, it can be safely assumed that the right to education is protected in the selected panchayats.

The panchayat members and beneficiaries concur on the presence of government primary schools within 1 km and government upper primary schools within 3 km of children's houses in a panchayat [E6]. They also agree that free textbooks and school uniforms are distributed to all beneficiaries [E7]. The beneficiaries fully endorse the claim of the panchayat members that the schools are equipped with modern infrastructural facilities (classroom, playground, benches and computer education) with safe and clean drinking water facilities and sanitation facilities [E8]. They affirm that the schools have separate toilets for female, male and disabled students [E9]. The responses of the panchayat members, when corroborated with the beneficiaries for the questions designed to measure obligation to “fulfill” right to education, show that right to education is being fulfilled.

However, the scores dip again when it comes to the questions designed to measure obligation to “promote”. Only half of the panchayat members assert that they run awareness campaigns about Samagra Shiksha Abhiyan [E10] and convene special Gram Sabha meetings for education [E11]. However, this is corroborated by only one-fifth of the beneficiaries. The low scores of panchayat members and beneficiaries indicate that the right to education is not being substantially promoted, thus hindering the realization of the right. While efforts to improve educational infrastructure and resources are commendable, the lack of effective communication regarding educational schemes to beneficiaries remains a significant barrier. While there is some effective promotion of educational rights, more proactive measures are needed to inform and engage the community about these opportunities. Improved communication strategies and outreach programs are crucial to bridge this gap and ensure that all community members are aware of and can benefit from educational initiatives. Research underscores the significance of community-based approaches in effectively promoting education (Sinha, 2005). Outreach programs and local engagement are critical for ensuring educational schemes reach their intended beneficiaries (Burnette et al., 2016).

Panchayati Raj Institutions can play a crucial role in realizing the right to education. To ensure substantial changes in school governance, Gram Panchayats must be active, well-informed and able to make independent decisions (Sadgopal, 2010). A method for role implementation by Panchayats must be devised to realize the right to education.

### Status of realization of the right to political participation

**Figure 3:**
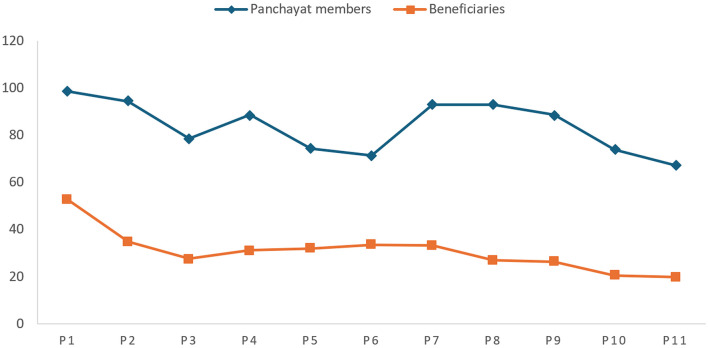
Status of right to political participation.

The survey questionnaire administered to the panchayat members and the beneficiaries contained 11 questions related to the right to political participation (PP). PP1, PP2, PP3 and PP4 were designed to measure the “respect” aspect, whereas PP5 and PP6 were intended to see whether the right to political participation was being protected. PP7, PP8 and PP9 were designed to measure the aspect of fulfillment of the right. PP10 and PP11 dealt with the obligation to “promote”. Figure 3 compares the average responses of panchayat members and beneficiaries.

Figure 3 shows that even though almost all the panchayat members claim to have run campaigns for encouraging voting, joining a political party, or standing for election [PP1], this is confirmed by only half of the beneficiaries. The scores of beneficiaries dip further over the question of regularity of Gram Sabha meeting [PP2], with barely one-third of the beneficiaries responding in favor. While 78% of the panchayat members claim to have facilitated meetings of villagers with the public officials [PP3], only 27% of the beneficiaries agreed. Even over the provision for peaceful protests in the panchayats [PP4], only 31% of the beneficiaries respond positively, whereas nearly 90% of the panchayat members assert the same. This indicates that there is a need for improvement on the obligation to “respect” the right to political participation. Research suggests that a specific group of villagers participate more in political activities than others. Such villagers are mostly males, well-informed and educated, while landless people, tribals and women participate less in the activities of panchayat (Alsop et al., 2001). It has been pointed out that the state does no more than facilitate and organize the elections; it doesn't ensure that villagers are informed about the intricacies involved in the elections and the democratic objectives behind such a process (Sharma, 2001).

Only about 70% panchayat members agreed that they have organized special campaigns for Scheduled Castes (SC), Scheduled Tribes (ST), women, minorities, lower caste and other marginalized people to participate in voting, joining a political party, or standing for elections [PP5], however this was corroborated by only one third of the beneficiaries. This trend remained the same for the next question, which related to organizing a special campaign for SC, ST, women, minorities, lower caste, and other marginalized people to participate in the Gram Sabha Meeting [PP6]. This shows that the right to political participation is not being substantially protected. Studies confirm that mere representation of women in the form of a reservation is not enough to bring qualitative change in local politics, and a lot needs to be done to create ground for a more inclusive form of grassroots governance (Mishra, 2018). Despite reservations for SC, ST and women in panchayats, instances of caste discrimination, harassment and violence are widespread for panchayat leaders belonging to these communities, thus hindering their right to political participation (Haokip and Umarani, 2018). Affirmative action has not helped ensure proper and effective representation of the disadvantaged and marginalized groups (Patnaik, 2005).

As one moves to the questions related to the obligation to “fulfill” the right to political participation, the scores of the beneficiaries continue to dip. Despite excellent responses from the panchayat members, the beneficiaries disclaim the same. Only 33% of the beneficiaries confirm the regular holding of Gram Sabha meetings [PP7]. Further, only 26% of beneficiaries agree that prior notice of such meetings is given out [PP8] and that the resolutions adopted in the meeting of Gram Sabha are implemented [PP9]. The low responses of beneficiaries indicate the need for the state to work on the “fulfill” aspect of the right to political participation. Gram Sabha meetings are not being held regularly, and this is due to a lack of involvement, a lack of awareness amongst elected representatives of PRIs, poor information communication, low attendance in the meetings and a lack of clear understanding amongst the people about the importance and the functions of the Gram Sabha (Datta, 2019).

The beneficiaries also dispute the organizing of a campaign for people to actively and regularly participate in Gram Sabha meetings for public policy making [PP10], as only 20% of the beneficiaries responded favorably. They also disclaim that issues related to political participation are raised in the Gram Sabha meeting [PP11]. This shows that even though the panchayat members are trying to ensure the realization of the right to political participation of the beneficiaries, their efforts have yet to reach the beneficiaries. Gram Sabhas continue to be marred by people who do not participate; their utility must be grasped by the panchayat members and the villagers (Nambiar, 2001).

Studies have highlighted that political participation in rural areas is often limited by a lack of awareness and engagement (Mansuri and Rao, 2013). Effective participation requires targeted efforts to educate and involve all community members. Promotion of the right to political participation is inadequate. The awareness and political participation gap underscores the need for more robust initiatives to encourage active participation. Better communication, education, and engagement strategies are essential to effectively promote the right to participation. Initiatives to increase political awareness, particularly among women and youth, are critical for ensuring inclusive and effective participation. Literature suggests that promoting political participation requires a multifaceted approach, including education, outreach, and capacity-building initiatives (Hickey, 2004). These strategies are crucial for fostering an inclusive political environment.

## Discussion

The study analyzes the status of realization of selected human rights, i.e., right to health, right to education and right to political participation, in terms of the “respect, protect, fulfill and promote” framework in the selected panchayats of Indian Punjab. These rights assume vast importance for the rural population. Education has the potential to empower individuals, drive economic development and foster social progress (Grant, 2017). The right to education allows individuals to change the status quo and break the intergenerational cycles of poverty and deprivation (Perry et al., 2006). The right to health and the implementation of health-related schemes in rural areas are of utmost importance to the rural population, which faces significant hurdles in accessing quality health care facilities in remote areas (Strasser et al., 2016). The remoteness of villages, lack of resources and huge out-of-pocket healthcare expenditure hinder the realization of the right to health at the panchayat level. There exist deep socio-economic and religious communal divides in Indian villages, which have the propensity to restrict the political participation of the vulnerable classes (Mitra, 1993). Realization of the right to political participation then assumes importance for scheduled castes, scheduled tribes, women, persons with disabilities and other marginalized communities so that their issues can come to the fore (Hasan, 2011).

### Right to health

Article 12 of the ICESCR recognizes the right to health (Chinkin, 2006). It proclaims that everyone has the right “*…to the enjoyment of the highest attainable standard of physical and mental health”* (MacNaughton and Hunt, 2009). Para 33 of General Comment 14 on the right to health states that right to health “*…imposes three types or level three types or levels of obligations on States parties: the obligations to respect, protect and fulfill”* (MacNaughton and Hunt, 2009). In relation to the right to health, the obligation to respect requires States to refrain from interfering with the enjoyment of the right to health (Leary, 1994). The obligation to protect requires states to prevent third parties from interfering with individuals' right to health (Gostin and Archer, 2007). The obligation to fulfill requires the States to fully adopt all necessary measures to realize the right to health (Yamin, 2005).

Right to health has not been recognized as a fundamental right under the Constitution of India (Srivastava, 2023). However, the constitutional courts of India have consistently read the right to health as implicit in the right to life guaranteed under Article 21 of the Constitution (Bhat, 2022). Further, no legislation has been brought providing statutory recognition to the right to health in India (Pandey, 2024). The government, however, has the National Health Policy 2017 in place and runs several healthcare schemes to aid public healthcare (Mohanan et al., 2016). National Rural Health Mission is one such scheme launched to provide healthcare facilities to the rural population (Husain, 2011).

The availability, acceptability, accessibility, and quality of healthcare products and services comprise the right to health (Zuniga et al., 2013). According to Article 24 of the United Nations Convention on the Rights of the Child, children have the right to the best possible standard of health and access to facilities for illness treatment and health rehabilitation (Whalen, 2022). Women are entitled to free and easily accessible health services related to pregnancy and postpartum care, as stated in Article 12 of the Convention on the Elimination of All Forms of Discrimination Against Women (Shalev, 2000). The objective of SDG 3 is to guarantee everyone's health and wellbeing, regardless of age (Das et al., 2021). The targets of SDG 3 include lowering the ratios of maternal and newborn deaths, putting an end to diseases and epidemics, preventing and treating substance abuse, lowering the number of fatal traffic accidents, providing universal access to sexual and reproductive health care, lowering the number of deaths and illnesses linked to pollution, and more (Buse and Hawkes, 2015). The Constitution of India recognizes the right to health as one of the Directive Principles of State Policy (Sharma et al., 2021). The State is required by Article 47 of the Constitution to take action to enhance public health (Srivastava, 2023). In alignment with the Sustainable Development Goals, the National Health Policy of 2017 aims to provide universal access to high-quality health care services and the best possible level of health and wellbeing for all people of all ages in India (Asgari-Jirhandeh et al., 2021).

On April 12, 2005, the National Rural Health Mission (NRHM) was established (Husain, 2011). The Mission aimed to offer rural communities nationwide easily accessible, reasonably priced, and high-quality healthcare (Bondale et al., 2014). Raising public health spending, building a strong health care infrastructure, reviving local health practices, integrating health issues with other determinants of health through decentralized management, addressing health care disparities, and enhancing rural residents' access to health care services—particularly impoverished women and children—were all part of the mission's vision (Dhingra and Dutta, 2011). In addition to the Village Health and Sanitation Committee, the NRHM planned for each village to have one Accredited Social Health Activist (ASHA) and one Anganwadi Worker (AWW) (Saprii et al., 2015). A sub-health center with two auxiliary nurse midwives (ANM) and one multipurpose worker was established at the Gram Panchayat level for five to six villages. A Primary Health Center with three staff nurses, an ambulance, and 24-h services was available to a group of Gram Panchayats, or 30–40 villages. At the block level (for 100 villages), a hospital was established that could handle obstetric and surgical medical emergencies at any time and offer 24-h services. In the hopes that community involvement would improve accountability and produce favorable outcomes, NRHM suggested accountability at all levels.

It has been reported that PRI engagement significantly improves healthcare workers' attendance, availability of medicines, quality of services offered, and infrastructure (John, 2012). Nanjunda (2020) has reported that the ineffective and non-participatory role of PRIs in decision-making has prevented the rural poor population from fully benefiting from NRHM. This has been linked to local Panchayats' complicated and disorganized processes and a failure to increase healthcare awareness. Kumar and Mishra (2016) have reported that PRIs' involvement in primary health care is fraught with problems, such as prioritizing service providers and users, engaging in unethical coercive behavior, and lacking communication. Nonetheless, there are certain benefits to adopting PRIs in service delivery, like improved consistency and availability of medical professionals at health centers (Kumar and Mishra, 2016).

Gupta (2010)s has reported that participation of panchayati raj institutions facilitates the timely delivery of high-quality medications and other critical medical supplies to health centers. Rajesh and Thomas (2012) found that decentralization has increased access to healthcare in rural areas by improving primary and secondary healthcare facilities' equipment and infrastructure. Malviya et al. (2014) reported that the Village Health and Sanitation Committee is still mostly unknown to many PRI and Self-Help Groups members. There has never been any formal training given to the members of these committees about how these committees function at the village level. These committees are crucial to health planning, but their efficacy is hampered by several factors, including irregular committee meetings, a lack of money, public interest, insufficient attention, and the unfair actions of Panchayati Raj officials (Kumar and Mishra, 2016). A lack of accountability, political party domination, and the absence of regular periodic elections hinder the capability of PRIs in general to improve health indicators and increase public awareness of health initiatives and other significant issues (Nanjunda, 2025).

This study's findings show a lack of awareness about the National Rural Health Mission scheme among the panchayat members and beneficiaries, leading to the inference that the right to health is not being substantially “respected” in the selected panchayats. Coming to the obligation to “protect”, both categories of respondents agree on the availability of medicines and health care services at the primary health care center. They also agree that the health officials and ASHA workers regularly perform their duties. Hence, it can be said that the right to health is being substantially “protected” at the selected panchayats.

Concerning the obligation to “fulfill”, even though the panchayat members affirm the availability of good infrastructure at the primary health care center and cleanliness in the panchayats, the beneficiaries refute the same. This shows the lack of satisfaction among the beneficiaries. Both panchayat members and beneficiaries show alarming awareness about VHSNC and its functioning, confirming the findings of the past studies. The analysis of their responses shows that the right to health is not being substantially fulfilled in the selected panchayats.

The disagreement of the panchayat members and the beneficiaries over the implementation of awareness programs about the NRHM and raising of issues related to the right to health at the Gram Sabha meeting shows that the right to health is not being substantially promoted in the selected panchayats.

### Right to education

Article 13 of the ICESCR recognizes everyone's right to education and proclaims that “*primary education shall be compulsory and available free to all”* (Sheppard, 2023). As per the General Comment No. 13 of the Committee on Economic, Social and Cultural Rights, the right to education, like other rights, imposes tripartite obligations on the states, i.e., the obligations to respect, protect and fulfill (Kalantry et al., 2010). The Committee observed as follows:

“*The obligation to respect requires States parties to avoid measures that hinder or prevent the enjoyment of the right to education. The obligation to protect requires States parties to take measures that prevent third parties from interfering with the enjoyment of the right to education. The obligation to fulfill (facilitate) requires States to take positive measures that enable and assist individuals and communities to enjoy the right to education. Finally, States parties have an obligation to fulfill (provide) the right to education. As a general rule, States parties are obliged to fulfill (provide) a specific right in the Covenant when an individual or group is unable, for reasons beyond their control, to realize the right themselves by the means at their disposal.”*

In India, the right to education has been guaranteed as a fundamental right under Article 21-A of the Constitution of India (Rao, 2008). The right to free and compulsory education of children aged six to fourteen has also been guaranteed via the Right of Children to Free and Compulsory Education (RTE) Act, 2009 (Singh and Nagpal, 2010). The Indian government launched the Sarva Shiksha Abhiyan scheme in 2001 to realize the right to education in India (Ward, 2011). Section 3 of the 2009 Act recognizes the right of a child aged six to fourteen years to free and compulsory education in a neighborhood school till the completion of elementary education (Dyer et al., 2022). The Act also lays down the duty of appropriate governments, local authorities and parents and guardians (Maheshwari, 2021). Section 13 of the Act prohibits the collection of a capitation fee or any screening procedure for admission by the schools (Kumar, 2022). Section 21 of the Act provides for the constitution of the School Management Committee consisting of “*...the elected representatives of the local authority, parents or guardians of children admitted in such school and teachers”* (Dyer et al., 2022). It also lays down the functions of the School Management Committee, which include monitoring the school's work, preparing the school development plan, and monitoring the utilization of grants received by the school.

All people should have free and unrestricted access to education, as per the right to education (Spring, 2000). Even Sustainable Development Goal 4 calls for promoting lifelong learning opportunities for everyone, acknowledging that the right to education should not be limited to a specific age group (Elfert, 2019). India implemented several government programs to achieve universal education. Still, the most notable is the Sarva Shiksha Abhiyan (SSA), which was renamed the Samagra Shiksha Abhiyan in 2018 (Sharma and Pattanayak, 2022).

In India, the Sarva Shiksha Abhiyan was started in 2001 to provide elementary education to all children between the ages of 6 and 14 by 2010 (Yadav et al., 2018). To actively incorporate the community in school management, SSA had defined three tiers for the community-based planning process: habitation, block, and district (Bhatty, 2022). Representing grassroots-level stakeholders and structures, including village education committees, panchayati raj institutions, community leaders, educators, and parents, was sought at the habitation level. Similarly, core planning teams comprising members from different departments and stakeholders were also envisioned at the block and district levels. Through the 86th Constitutional Amendment, 2002, the right to education was acknowledged as a fundamental right under Article 21A of the Indian Constitution (Rao, 2008). However, the amendment and the Right of Children to Free and Compulsory Education (RTE) Act, 2009, did not go into effect until April 1, 2010. A child became entitled to the right to free, compulsory education at a neighborhood school due to these legislative reforms.

Panchayati Raj institutions are essential to advancing education (Parashar and Kumar, 2017). Panchayats at the village, block, and district levels must be reinforced and involved in the planning and administration of elementary education for the RTE Act, 2009, to be adequately implemented (Tyagi, 2012). Programs for educational reform and the universalization of basic education can be significantly aided by the School Management Committee, which comprises the headmaster, teachers, parents/guardians, and elected local representatives (Bhattacharya and Mohalik, 2015). To guarantee significant changes in school governance, Gram Panchayats must be proactive, well-informed, and capable of making decisions independently. Without exception, the long-held objective of giving all children free, high-quality elementary education would be aided by the participation of panchayats with constitutional standing; however, no concrete strategy for the role implementation by panchayats has been developed in this regard (Kumar, 2015).

The present study's findings show a need to improve upon the obligation to “respect” as beneficiaries lack awareness about the Samagra Shikha Abhiyan scheme and its entitlements. While the panchayat members reported a good understanding of the schemes, the response of the beneficiaries remained dismal. However, in the case of the obligation to “protect” and “fulfill”, the reactions of the panchayat members have been excellent and have even been substantially corroborated by the beneficiaries. They have reported in the affirmative that there are equal educational opportunities for all students, no discrimination among children from various communities, and a safe environment for female students and staff within the school premises. Even in the case of the obligation to “fulfill”, both classes of respondents concurred on the presence of schools at a reasonable distance with free distribution of books and uniforms. The panchayat members asserted that the schools were equipped with facilities suitable for learning, which was affirmed by the beneficiaries.

However, the responses of the elected representatives and the beneficiaries dipped again when it came to the questions related to the obligation to “promote”. The low reaction by beneficiaries over the questions related to awareness campaigns about Samagra Shiksha Abhiyan and convening of special Gram Sabha meeting for discussing issues related to education showed that the panchayats are lagging in the obligation to “promote”.

### Right to political participation

The ability to directly or indirectly engage in state political activities is facilitated by the right to political participation (Isin and Turner, 2002). Everyone has the right to participate in their nation's governance, directly or indirectly, through representatives they have freely selected, according to Article 21 of the UDHR (Suksi, 2002). According to Article 25 of the International Covenant on Civil and Political Rights (ICCPR), everyone is entitled to participate in public affairs, vote in legitimate periodic elections, and be elected, without unjustified limitations (Abdulai, 2023). According to Article 19 of the UDHR and ICCPR, people can hold and express their opinions (O'Flaherty, 2012). Goal 16 of the Sustainable Development Agenda, which advocates for peace, justice and strong institutions, is linked to the right to political participation (Monaco, 2024).

The Gram Sabha, a village assembly of all the adults, debates the Gram Panchayat's development work plans, known as the Gram Panchayat Development Plan (GPDP) (Mohapatra, 2020). The elected panchayat members then carry out the plans. The efficiency of public services is increased with the creation of GPDP. Ensuring excellent governance is the Gram Panchayats' responsibility. The PRI is a tool for maintaining the “consensus-oriented” and “participation” elements essential to good governance. The approach employed in this context is a bottom-up strategy that aims to align with the requirements of diverse stakeholders (Raj, 2015). The functioning of the Gram Sabha and Gram Panchayats served as the foundation for analyzing the right to political participation in this study.

In Punjab, women have become much more involved in voting in recent years, although they have been relatively under-represented in other political activities. When exercising their right to political participation, women in Punjab encounter psychological, sociocultural, and political obstacles (Kaur and Singh, 2021). Affirmative action in decentralization has failed to ensure adequate representation of underrepresented groups, despite the potential for inclusion and empowerment (Patnaik, 2005). While women's political engagement in panchayat elections is generally excellent, their political indifference is evident in assembly and parliamentary elections. The poor level of education, male-dominated culture, and society all have a significant role in the backwardness of women (Sahoo, 2019; Kaul and Sahni, 2009 and Panday, 2013).

The findings of this study indicate that the right to political participation is not being substantially respected in the panchayats. Only half of the beneficiaries agree that campaigns encourage them to contest elections and exercise their right to political participation. The beneficiaries‘ responses remain dismal over the regularity of Gram Sabha meetings and panchayat members facilitating their meetings with public officials. The scores continue to dip for the questions related to the obligation to “protect” as well. The beneficiaries' responses show that they are not satisfied with the efforts of panchayat members for ensuring effective political participation of Scheduled Castes (SC), Scheduled Tribes (ST), women, minorities, lower caste and other marginalized people. Thus, it can be inferred that the right to political participation is not substantially protected at the selected panchayats.

The beneficiaries‘ responses to the questions related to the obligation to “fulfill” indicate the apathy of panchayat members toward the right to political participation. The panchayat members assert that they hold Gram Sabha meetings regularly or that prior notice of such meetings is given, but the beneficiaries' responses refute such claims. Right to political participation is also not substantially promoted in the panchayats, as many beneficiaries deny that campaigns for encouraging political participation are regularly held. They also deny that such issues were raised during the Gram Sabha meeting.

## Conclusion

The significance of local self-government institutions for implementing human rights cannot be overemphasized. Their proximity to the people makes them ideal institutions for realizing human rights. The Gram Panchayats are conceived as an intermediary for government schemes in villages, and their representatives are often tasked with facilitating the benefits for their beneficiaries. The present study highlights that even though there are laws and schemes to enable the realization of rights by the masses, there remain gaps in the implementation thereof. The “respect”, “protect”, “fulfill” and “promote” framework utilized in this study offers insights into the degree to which the state obligation concerning the selected human rights is being upheld and how the variation can be explained. This study is a novel attempt to improve the understanding of the ideal conditions for realizing human rights. The findings show that even when the state creates machinery for implementing human rights, violations may still occur, and progressive improvement on such obligations is much needed. The study shows disparities between the claims of the panchayat members and the beneficiaries' experience, highlighting gaps in the realization of human rights in rural areas. The study proposes that the governmental policies intended to realize human rights must be formulated as per the respect, protect, fulfill and promote framework.

## Data Availability

The datasets generated by the survey research are available in the Zenodo repository: https://doi.org/10.5281/zenodo.14602983.
